# The Connection between the Toxicity of Anthracyclines and Their Ability to Modulate the P-Glycoprotein-Mediated Transport in A549, HepG2, and MCF-7 Cells

**DOI:** 10.1155/2014/819548

**Published:** 2014-01-19

**Authors:** Aneta Rogalska, Marzena Szwed, Błażej Rychlik

**Affiliations:** ^1^Department of Thermobiology, Faculty of Biology and Environmental Protection, University of Lodz, Pomorska 141/143 Street, 90-236 Lodz, Poland; ^2^Department of Molecular Biophysics, Faculty of Biology and Environmental Protection, University of Lodz, Pomorska 141/143 Street, 90-236 Lodz, Poland

## Abstract

Multidrug resistance (MDR) is a major obstacle to the successful chemotherapy of solid tumors. We compared the resistance of the most popular solid tumors, breast adenocarcinoma (MCF-7 cell line) and nonsmall cell lung (A549 cell line) hepatocellular liver carcinoma (HepG2 cells), to aclarubicin (ACL) and doxorubicin (DOX). This research aimed at determining the relation between the toxicity of ACL and DOX, their cell accumulation, and then effect on P-glycoprotein functionality. ACL is more cytotoxic for tumor cells compared to DOX. The intracellular concentration of drugs in cancer cells was dependent on the dose of the drugs and the time of incubation. The P-gp inhibitor Verapamil (V) increased DOX accumulation in all tested cell lines. By contrast, the intracellular level of ACL was not affected by this modifying agent. The assessment of the uptake of 5,5′,6,6′-tetrachloro-1,1′,3,3′-tetraethylbenzimidazolocarbocyanine iodide (JC-1) or Rhodamine 123 (R123) allows the evaluation of the different influence of drugs on P-gp activity which is in agreement with the estimation of expression measured by MDR-1 shift assay. These data suggest that ACL is less P-gp dependent than DOX and consequently may be used in a clinical setting to increase treatment efficacy in resistant human tumors.

## 1. Introduction 

Anthracyclines are potent anticancer agents which have been used in the treatment of acute leukemias, Hodgkin's disease, sarcomas, and solid tumors. Despite their generalized application for more than 40 years, the mechanisms of anthracyclines cytotoxicity have long been a matter of controversy. Continuous application of anthracyclines, especially doxorubicin (DOX), led to the development of severe heart failure [1]. The cardiotoxic effect of anthracyclines may affect irreversible and incurable cardiomyopathy, which impairs the quality of life and increases the risk of death [2]. Besides this, the therapeutic activity of anthracyclines is lower that in the case when tumor cells are resistant to anticancer drugs application. Multidrug resistance is the main cause of anticancer treatment failure. Several data have been published regarding various cellular mechanisms of drug resistance [3, 4]. One of the most important cellular transporters is P-gp [5, 6]. This protein was first discovered in mammalian cells that had been selected for resistance to the drug [7]. Several anticancer drugs may be removed from neoplastic cells by P-gp-mediated transport, despite the diversity in their chemical structures and mechanisms of action [8].

Aclarubicin (ACL) is one of the newest N,N-dimethylated trisaccharide anthracyclines with aclavinone aglycone. The main mechanisms of its action are similar to other anthracyclines, but there are a few differences. ACL efficiently binds to DNA, leading to a secondary inhibition of the catalytic activity of topoisomerase II (topo II) and enhancement of the concomitant poisoning effect on topo I [9]. During clinical application, aclarubicin is shown to be active and cardiac-tolerable in adult patients with acute myeloblastic leukemia [10, 11]. Moreover, aclarubicin induced late cardiac events in a phase II study of adult patients with refractory acute myelogenous or lymphoblastic leukemia [12]. ACL can also be used as a single agent or in combination with other anticancer drugs. After remission, patients with acute myeloid leukemia (AML) received high-dose cytarabine (HD-Ara-C), a DNA-synthesis blocker, in combination with mitoxantrone, etoposide, or aclarubicin as a postremission treatment, which improves long-term disease-free survival [13].

It has been suggested that the ability of drug molecules to be recognized and transported by P-gp can be related to the degree of resistance to these molecules and compounds that are poor substrates for P-gp are efficient cytostatic agents against MDR cells [14]. A number of original papers show that the 9-alkyl substitution of the anthracene A ring and certain sugar modifications of classic anthracyclines have been associated not only with toxicity but also with reduced affinity for P-gp and the maintenance of cytotoxic activity in MDR tumor cell lines [15, 16].

In this study, we aimed to investigate how far aclarubicin weakens the function of the P-gp transport system and what is the relation between the toxicity of this anthracycline and P glycoprotein functionality after drug treatment. All experiments were performed on three cell lines: breast adenocarcinoma (MCF-7), nonsmall cell lung (A549), and liver hepatocellular carcinoma cell line (HepG2) in the absence and presence of the calcium channel blocker, Verapamil [17]. The cell lines were chosen as representatives of the cancers, most common globally at present. Our results suggested that ACL, in contrast to DOX, was more cytotoxic to tested tumor cell lines, probably due to their different accumulation and distribution. Such variety in DOX and ACL toxicity can be associated with their cellular retention which influences the level of intracellular drug content. These data can support the idea that the role of P-glycoprotein is more significant in the amount of doxorubicin expelled out of the cell than in the amount of aclarubicin transported outside the cell.

## 2. Materials and Methods

### 2.1. Chemical Products

ACL and DOX were obtained from Sequoia Research Products (Pangbourne, UK). Dulbecco's Modified Eagle's Medium (DMEM) was supplied by Cambrex (Basel, Switzerland), fetal bovine serum and trypsin-EDTA were from Gibco (Edinburgh, Scotland). JC-1, R123 and Verapamil were purchased from Sigma (St. Louis, USA). The MDR shift assay was supplied by Calbiochem. All other chemicals and solvents were of high analytical grade and were obtained from Sigma or POCH S.A. (Gliwice, Poland).

### 2.2. Cell Lines

The cell lines used in this study were A549, HepG2, and MCF-7 derived from solid tumors. They were a kind gift from the Department of Molecular Biophysics of the University of Lodz and from the Laboratory of Transcription Regulation of the Medical Biology Center of the Polish Academy of Sciences, Lodz, Poland. Cells were cultured in tissue culture dishes at 37°C, 5% CO_2_ in DMEM containing 10% fetal bovine serum. The typical density of cells used in the experiments was as follows: for measuring the intracellular ac6cumulation of drugs it was 2 × 10^5^ cells/dish, and for a functional assay of P-gp and an immunofluorescence assay of P-gp expression 10^6^ cells/mL.

Exponentially growing cells with viability higher than 95% were subjected to aclarubicin and doxorubicin treatments.

### 2.3. Cytotoxicity Studies

The effect of doxorubicin and aclarubicin on A549, HepG2, and MCF-7 cell growth was determined by using MMT assay (3-(4,5-Dimethylthiazol-2-yl)-2,5-diphenyltetrazolium bromide) in 96-well microtiter plates with float-bottomed wells (Nunc) in a total volume of 200 *μ*L. Cells at a density of 1 × 10^4^ cells/well were treated with DOX (0.5–12 *μ*M) or ACL (0.01–8 *μ*M) for 2 h, the medium was replaced with drug-free medium, and the cells were cultured for a further 72 h. Then, MTT was added for 4 h. Formazan crystals, formed by mitochondrial reduction of MTT, were solubilized in dimethyl sulfoxide, and the absorbance was measured at 570 nm using a microplate reader (Awareness Technology Inc., USA). The IC_50_ parameter, defined as the drug concentration that reduced cell growth to 50% of the control cells, was calculated from the linear transformation of the dose-responses curves.

In order to examine the effects of Verapamil on the DOX or ACL cytotoxicity, the cells were preincubated with this P-gp inhibitor (30 *μ*M) for 30 min. The dose of Verapamil concentration was evaluated during assessment of its toxicity on the tested cell's viability.

### 2.4. Analysis of Intracellular Accumulation of Anthracyclines

The intracellular accumulation of ACL and DOX in A549, HepG2, and MCF-7 cell lines was measured by flow cytometry (LSRII, BD Biosciences). The drugs excitation and emission wavelengths were determined experimentally by using Cary 50 spectrofluorometer (Melbourne, Australia). The cells were incubated with the IC_50_ of each anthracycline for 2 h under normal cell culture conditions. Then, the cells were removed from the culture dishes by trypsinization and centrifuged and suspended in ice-cold PBS. To show the effect of P-gp inhibitor on the anthracycline drugs accumulation A549, HepG2, and MCF-7 cells were preincubated with Verapamil (30 *μ*M) for 30 min and then incubated with IC_50_ values of ACL or DOX for 2 h. In another experiment, cells, after 2 h incubation with the drugs, were washed with PBS at room temperature and then cultured in fresh medium for additional 24 h. Cellular drug fluorescence was measured using an argon laser at FL2 channel. For all samples 30 000 cells were counted and the analysis was performed using flow cytometer. Each experiment was repeated at least 3 times.

Furthermore, the intracellular accumulation and distribution of drugs were monitored by fluorescence microscopy (Olympus IX70, Japan) equipped with the NB filter (470–490 nm).

### 2.5. Evaluation of P-gp Activity

For detection of P-gp function as a transporter of cellular membrane, Rhodamine 123 and JC-1 were used as a fluorescence probe. The experiments were carried out using the following sequence. Cells seeded on Petri dishes at a density 10^6^ cells/mL were preincubated without or with 30 *μ*M Verapamil for 30 min at 37°C in medium; then ACL or DOX at IC_50_ concentration was added and the samples were further incubated for 2 h. After drug treatment the medium was removed, the cell monolayer was washed twice with PBS, and then the cells were removed from the culture dishes by trypsinization. After centrifugation, the cells were suspended in ice-cold PBS, and to all samples 50 *μ*L of R123 or JC-1 was added to a final concentration of 0.1 M. The cellular uptake of R123 and JC-1 was immediately analyzed on a flow cytometry (LSRII, BD Biosciences), equipped with a 488 nm argon laser and 2 fluorescence detectors: FL1 and FL2 channels.

### 2.6. Immunofluorescence Assay of P-gp Expression

The expression of P-gp was estimated using an immunofluorescence assay (MDR Shift Assay) according to the manufacturer's protocol. The mouse monoclonal antibody against human MDR-1 (UIC2 mAb) reacts with the extracellular moiety of P-gp and inhibits the P-gp-mediated efflux of the majority of the tested chemotherapeutic drugs. It has been shown that the reactivity of UIC2 mAb with P-gp was enhanced in the presence of P-gp substrates due to protein conformational changes [18, 19]. In our studies, cells were trypsinized, washed twice in PBS, and divided into four tubes (10^6^ cells/tube in 1 mL). The cells were then warmed at 37°C for 10 min and the tested substances were added, 5 *μ*L of vinblastine stock solution (4.5 mM in DMSO; C-positive control) to the first tube, while the second tube contained 5 *μ*L of DMSO (B-negative drug control), and the third and the fourth tubes were incubated with IC_50_ doses of ACL or DOX, respectively. According to the manufacture's procedure, samples were incubated for 10 min at 37°C. A phycoerythrin (PE) labeled antibody UIC2 (UIC2 PE) was added to each tube. After 15 min incubation at 4°C the cells were washed twice with ice-cold PBS and kept on ice until analysis. A PE labeled irrelevant anti-mouse antibody (mAb of IgG2a) isotype was used as a negative antibody control (A). The cells were analyzed by flow cytometry (LSRII, BD Biosciences) using FL2 channel.

### 2.7. Statistical Analysis

The data are expressed as a mean ± SD. An analysis of ANOVA variance with a Tukey post hoc test was used for multiple comparisons. All statistics were calculated using the STATISTICA program (StatSoft, Tulsa, OK, USA). A *P* value of < 0.05 was considered significant.

## 3. Results

### 3.1. Cytotoxicity Assay

**Figure 1:**
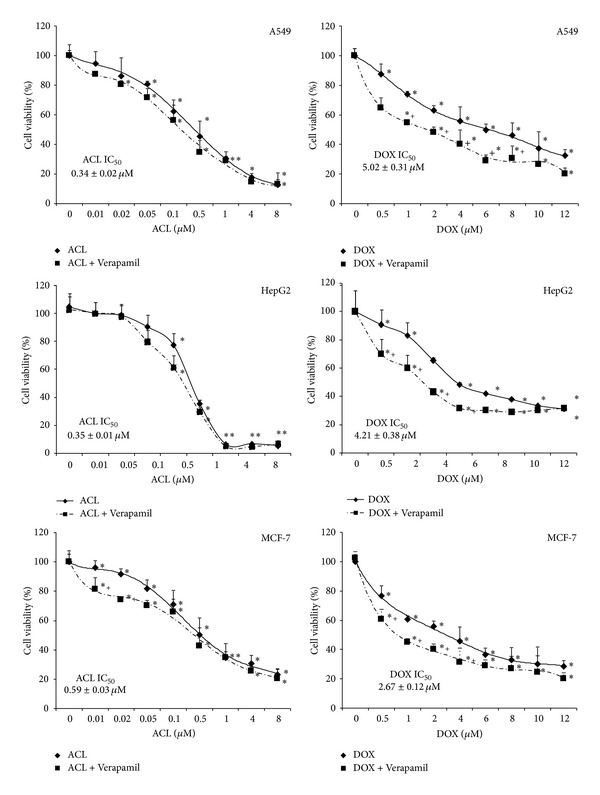
Representative dose-response curves following exposure to DOX and ACL of A549, HepG2, and MCF-7 cells. The evaluation of cell survival was assessed by MTT assay. *P < 0.05, the effect of DOX and ACL on the viability of solid tumor cells. ^+^ *P* < 0.05, changes between the probes preincubated with Verapamil. The values are the mean ± SD of four independent experiments.

The growth inhibitory effect of doxorubicin and aclarubicin against A549, HepG2, and MCF-7 and human cancer cells is shown in Figure 1, which presents the viability curves of analyzed solid tumor cell lines treated with ACL or DOX. The investigated cell lines exhibit a significantly different sensitivity to doxorubicin and aclarubicin. In each case, a marked decrease in cell viability with increasing drug concentration was observed. Our study demonstrated that ACL is a much more cytotoxic drug, about 14 times in A549, 12 times in HepG2, and 4.5 times in MCF-7 cell lines than DOX.

An additional variant of the experiment was the measurement of cell viability after exposure for 72 h to Verapamil at 0.5–100 *μ*M concentration range. We noticed that the effect of different Verapamil concentration was not statistically significant, and the viability of cells treated with Verapamil did not decrease below 90% (unpublished data). Therefore, to further the analysis, we used 30 *μ*M concentration of Verapamil, which is in agreement with many research articles. In probes preincubated with the Verapamil and then treated with DOX or ACL, we observed a considerably wide range of effects of these agents on cytotoxicity of ACL or DOX (Figure 1). Interestingly in A549, HepG2, and MCF-7 doxorubicin-treated cells Verapamil reduced the viability of DOX-treated cancer cells by about 25%. However, if the cells were preincubated with the P-gp modulator, a significant increase of ACL toxicity as was observed in cells treated with DOX was not noticed.

### 3.2. Accumulation of Drugs in the Tested Cells

**Figure 2:**
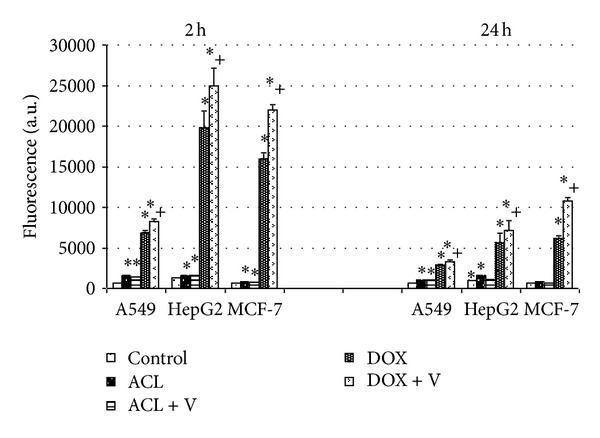
The effect of Verapamil on ACL and DOX accumulation in tested cells. The accumulation of ACL and DOX in A549, HepG2, and MCF-7 cell lines was examined after 2 h incubation with IC_50_ of the drugs and 24 h after incubation without drugs. The excitation and emission wavelengths were for ACL *λ*_ex_ = 436 nm and *λ*_ex_ = 585 nm and DOX *λ*_ex_ = 488 nm and *λ*_em_ = 566, respectively. *Values are statistically significant in comparison to the control, untreated cells, *P* < 0.05. ^+^Values are statistically significant in comparison to the cells incubated without Verapamil, *P* < 0.05.

As shown in Figure 2, the intracellular accumulation of ACL and DOX was dependent on the type of cell line and time of incubation. After a 2 h exposure the cellular ACL level in all tested cancer cells was significantly lower than that of DOX. However, following a 24 h posttreatment period the DOX accumulation was lower in A549, HepG2, and MCF-7 cells, 2.7, 3.5, and 2.8, respectively, in comparison to the cells treated for 2 h with drug. Under the same experiment the intracellular ACL level was markedly reduced in A549 cells only.

Figure 2 illustrates also intracellular accumulation of both drugs in the cell lines preincubated with P-gp modulator. In the presence of Verapamil, the most significant increase in the intracellular DOX accumulation during 2 h incubation with drugs was observed in MCF-7 (21972.17 a.u.) as well as in HepG2 (24990.5 a.u.). It is worth noting that in the case of cells incubated with DOX for 24 h we observed apparent differences of fluorescence intensity between the samples preincubated with Verapamil and probes treated with doxorubicin alone (in A549: 494.9; in HepG2 1654.3 and in MCF-7: 4598.7 a.u.).

**Figure 3:**
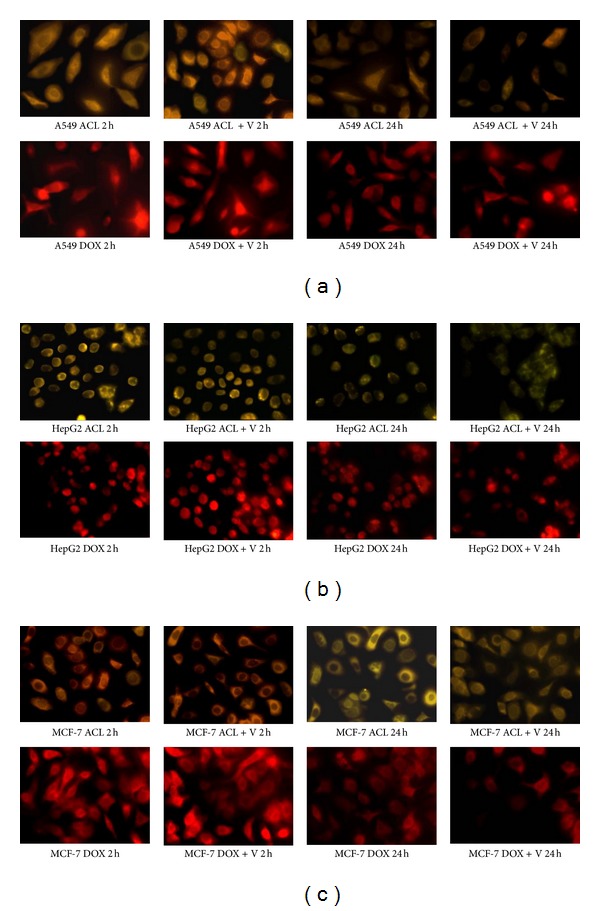
Intracellular DOX and ACL accumulation and their distribution in (a) A549, (b) HepG2, and (c) MCF-7 cell lines. The cells were incubated with IC_50_ of each anthracycline for 2 h, or, after 2 h incubation with the drugs, the medium was removed and the cells were cultured in fresh medium for another 24 h. In the experiments with Verapamil the cells were preincubated for 30 min at 37°C and then incubated with drugs. Fluorescence was monitored using an Olympus IX70, Japan, magnification 400x.

Differences in drugs accumulation were also analyzed by fluorescence microscopy.  Figure 3 shows the intracellular distribution of both drugs in the presence and absence of Verapamil in A549, HepG2, and MCF-7 cells where it was found that DOX was preferentially accumulated in the nucleus. In the cell lines treated with ACL, the fluorescence intensity was much weaker and it was detected mainly in the cytoplasmic vesicles. Stronger fluorescence was emitted, as expected, from the cells exposed to DOX and Verapamil. These findings also indicated that Verapamil considerably increased the DOX accumulation in the investigated cancer cells, whereas it did not change the intracellular level of ACL.

### 3.3. Functional Assay of P-gp

JC-1 and R123 were initially used as fluorescence probes for the analysis of the mitochondrial transmembrane potential [20]. However, these compounds are also successfully used for the analysis of P-gp activity. It was shown that not only R123 but also JC-1 is a substrate of P-gp and that the intracellular concentration of JC-1 depends on the activity of this transmembrane efflux protein [18].

**Figure 4:**
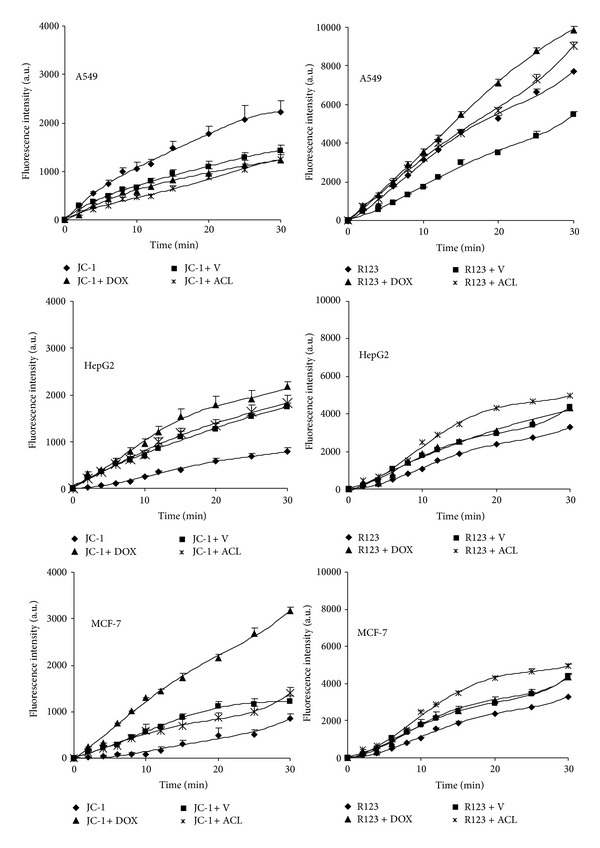
Uptake of R123 (right panel) or JC-1 (left panel) in the presence of Verapamil, DOX, and ACL by A549, HepG2, and MCF-7 cells in function of time. The values are the mean ± SD of four independent experiments.

In this study, we examined the effect of drugs on P-gp transport activity by measuring the intracellular accumulation of JC-1 and R123. The received results we compared with positive control, which was Verapamil pretreated samples. On the basis of the results presented in Figure 4, fluorescence ratios were calculated for each tested probe and presented as percentage values.

Figure 4 shows the time course of R123 and JC-1 uptake in the investigated cell lines. The percentage of intracellular fluorescence probes accumulation was presented in Table 1. The results indicate that the fluorescence intensity of both P-gp substrates increased with the increased time of the experiments. We observed the growth of accumulation of R123 and JC-1 in all DOX treated cell lines. In A549 cell line doxorubicin enhanced the uptake of probes (129.7% and 92.0% for R123 and JC-1, resp.) much more than positive control samples treated with Verapamil. P-gp functional activity did not change after ACL treatment in A549 cell line. MCF-7 cells expressed MDR-1 protein to a far greater level then non-small cell lung cancer cells. Therefore, the changes of P-gp activity in breast adenocarcinoma cells were easier to detect. The markedly higher accumulation of fluorescence probes was observed in HepG2 cells. Both anthracyclines in these cells improved R123 and JC1 cellular influx but aclarubicin caused a more major impact on the P-gp functionality than DOX. The relative assessment of P-gp activity obtained from the cytometric analysis in MCF-7 cell line indicates that both ACL and DOX modify the functionality of P-glycoprotein. However, doxorubicin can increase the accumulation of R123 and JC-1 by even 1.5 and 3.6 times, respectively, in comparison with positive control samples pretreated with Verapamil. The finding of the higher enhancement of P-gp activity in the presence of DOX was confirmed in all three tested cell lines.

### 3.4. Detection of MDR1 Expression

**Figure 5:**
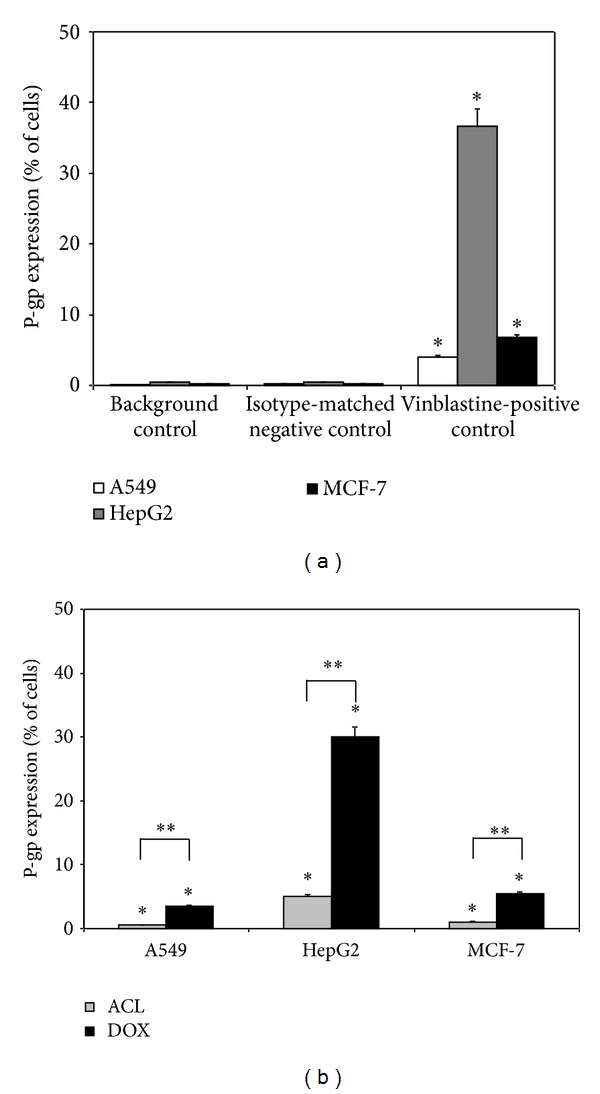
Analysis of the P-gp expression in solid tumor cell lines using vinblastine as positive control (a) and comparison of the effect of ACL and DOX on P-gp expression (b) in drug treated cells. *P < 0.05, significant differences between drugs treated and control cells. **P < 0.05, significant differences between cells treated with ACL and DOX.

To confirm the presence of P-gp in the tested cancer cells, immunofluorescence analysis was performed. Using monoclonal antibody UIC2, the highest expression of P-gp was detected in HepG2 cell line. As shown in Figure 5(a), the level of P-gp expression in MCF-7 and A549 cell line was lower in comparison with HepG2 cells. In cells exposed to anthracycline drugs the highest P-gp expression was found in DOX-treated HepG2 cells (30%). In contrast ACL did not significantly increase the expression of P-gp in all tested cancer cells lines (Figure 5(b)). Taken together, our results indicate the different effects of ACL and DOX on the P-gp activity in solid cancer cells. Our study suggests that ACL is a poor substrate of this transporter.

## 4. Discussion

In this study whether toxicity of common chemotherapy anthracyclines (ACL and DOX) is related with their cell accumulation and P-glycoprotein functionality was investigated. For this purpose, using solid tumor cancer cell lines (A549, HepG2, and MCF-7), we demonstrated that they are more sensitive to ACL treatment than to DOX. An analysis of the intracellular drug distribution indicated that DOX was located mainly in the nuclear fraction, whereas the presence of the aclarubicin was observed in the cytosol. The observed differences in the cytotoxicity and intracellular content of ACL and DOX might be related to their chemical structure and range of influences on the multidrug-resistant proteins [15]. The presence of the additional sugar residues makes aclarubicin more toxic, which implicates a more rapid cellular uptake and higher intracellular aclarubicin accumulation [21].

It is clear that the cytotoxic effect of anticancer drugs is connected with the dose which can be accumulated in the cells. The IC_50_ of ACL was significantly lower than that of DOX and the mean ratios of IC_50_ values for A549, HepG2 and MCF-7 were 2.3, 3.7, and 10, respectively. Furthermore, this toxicity of ACL and DOX is strictly correlated with induction of apoptosis and reactive oxygen species production [22].

However, in our research, we carried out preincubation of cells with Verapamil and we observed an increase in the effect of doxorubicin cytotoxic. Therefore, we tried to find the relation between the amount of drug which was transported to the cell and its toxicity. Presently, we noted that ACL was accumulated for a longer period of time than DOX. After 24 h of postincubation without drug in the medium, a decreased level of ACL was observed only in A549 cell line. A significantly lower intensity of DOX fluorescence was observed in all tested cell lines. We suggest that one of the most important reasons responsible for the decline of the intracellular level of the tested drugs is their active efflux from the neoplastic cells by multidrug transporters. P-gp is one of the most important multidrug transporters especially in HepG2 [23, 24] and MCF-7 cell lines [25, 26]. Our results are in agreement with Lehne et al. [15], who compared four anthracyclines with respect to their intracellular accumulation using a human hepatoma cell line rich in P-gp (HB8065 R) and a P-gp poor parental line. As a modifying agent, SDZ PCS 833 was used, which enhanced the accumulation of Epirubicin (EPI), DOX, and Daunorubicin (DNR) but not ACL. Similarly to the above mentioned authors, we did not observe any changes in the ACL intracellular level in the presence of Verapamil, in contrast to DOX. Therefore, we studied the functionality of P-gp in A549, HepG2, and MCF-7 cell lines and consequently assessed the activity of this protein after ACL and DOX treatments. In our research we used R123 and JC-1 fluorescent probes, which are known as substrates for P-gp, and evaluated the influence of ACL and DOX on their intracellular transport. We observed an increase of P-gp activity in all tested cell lines incubated with DOX. Our research also indicates that ACL does not modulate the functionality of P-gp especially in A549 cells. The studies are in agreement with experiments of drug efflux conducted by Lo et al. (2005) on the A549 cell line. They showed no difference between the influence of the P-gp inhibitors, Verapamil, or PSC 833 on R123 intracellular accumulation. Consequently, Lo and coauthors suggested that the expression of the multidrug resistance gene was not responsible for differences in cell sensitivity to ACL [26].

To confirm the presence of P-gp in the tested cancer cell lines we evaluated the P-gp expression using the MDR Shift Assay. UIC2 antibody recognizes and strongly inhibits P-gp-mediated efflux and reduces the resistance of MDR cells. The inhibitory effect of mAb (UIC2) is as strong as Verapamil at its highest clinically achievable concentration [27]. We observed P-gp expression in all the tested cell lines, even in A549 cell line treated with ACL. We suggest that immunohistochemical assay was a more sensitive method for detection of P-gp than functional assays. Just as in the case of the analysis of P-gp activity, the highest level of P-gp expression was noted in HepG2 cell line. Many experimental articles have also confirmed the presence of P-gp in the A549 cell line exposed to anthracycline drug, amrubicin [28, 29]. There is also a well-known correlation between the expression of P-gp and glutathione S-transferase in DOX-treated NSCLC [30]. The confirmation for this statement was the assessment of P-gp expression in two lung cell lines, the bronchiolar adenocarcinoma cell line (Calu-3), and A549 cells [31]. On the other hand, in 72 cases of advanced NSCLC, the scientists observed a breast cancer resistance protein (BCRP) expression but not P-gp, MRP1, MRP2, or MRP3 [32]. Consequently, Merk et al., 2011, suggest the implicative role of the lung resistance protein (LRP) in mediating resistance in lung cancer cells. All these features can cause poor clinical results for these tumors [33].

In our research, among the examined cell lines, the highest level of P-gp expression was observed in HepG2 cells. We suggest that ACL is not transported by P-gp and retains its activity in a multidrug-resistant human hepatoma cell line. ACL may overcome drug resistance due to an altered expression of topoisomerase II and glutathione S-transferase [34, 35]. Akimoto et al. report that the expression of P-gp in hepatocellular carcinoma was significantly correlated with the therapeutic outcome [36]. In liver cancer cells, BCRP and LRP proteins were detected as well [37]. These MDR proteins were also identified in MCF-7 cells [38, 39]. This may be the reason for the low activity of P-gp which was detected by our group. However, Cripe et al. suggest that P-gp is one of the proteins which might have a predictive value for the clinical outcome of breast cancer [40]. Thus, there is a strong correlation between the degree of P-gp expression and resistance to DOX in MCF-7 cells [41].

In conclusion, the findings of our study provide evidence that there is a connection between the toxicity of ACL and DOX and their accumulation in human solid tumor cells. DOX, which indicates lower toxicity, leads to a higher expression of P-gp than ACL. Additionally, the strong cytotoxic properties of ACL may be associated with its lower affinity to P-gp. Therefore, we suggest that the level of P-gp expression is a useful principle, but not the only one, for evaluating multidrug resistance in ACL treated solid tumor cells.

## Figures and Tables

**Table 1:** Flow cytometry analysis of P-gp functional activity with R123 or JC-1 in A549, HepG2, and MCF-7 cells.

Sample	Intracellular accumulation of JC-1 and R123 (%)		
	A549	HepG2	MCF-7
R123	100.0 ± 5.1	100.0 ± 7.2	100.0 ± 6.2
R123 + V	70.6 ± 7.2	124.89 ± 3.7*	110.8 ± 3.9
R123 + DOX	129.7 ± 3.6*	126.88 ± 9.2*	148.2 ± 8.3^∗+^
R123 + ACL	111.2 ± 5.9	157.29 ± 6.5^∗+^	137.7 ± 11.7^∗+^
JC-1	100.0 ± 2.9	100.0 ± 4.7	100.0 ± 8.6
JC-1 + V	85.2 ± 5.6	212.41 ± 5.2*	161.2 ± 8.9*
JC-1 + DOX	92 ± 5.5	250 ± 2.9^∗+^	386.8 ± 13.3^∗+^
JC-1 + ACL	86.9 ± 13.9	212.59 ± 7.0^∗+^	158.3 ± 14.6*

Results represent means ± SD of 4 independent experiments. *P < 0.05, significant differences between P-gp functional activity calculated from influx of R123 or JC-1 and R123 or JC-1 plus Verapamil and from R123 or JC-1 plus ACL or DOX after a 30-minute period of time monitoring the transport of fluorescence probes to the cells. ^+^ *P* < 0.05, significant differences between positive control samples (cells preincubated with Verapamil) and cells treated with DOX or ACL. P-gp functional activity was evaluated based on the basis of fluorescence ratios of R123 or JC-1 (Figure 4) and calculated for each tested probe. The final results were presented as percentage values.
